# The human cytomegalovirus terminase complex as an antiviral target: a close-up view

**DOI:** 10.1093/femsre/fuy004

**Published:** 2018-01-18

**Authors:** G Ligat, R Cazal, S Hantz, S Alain

**Affiliations:** 1 Université Limoges, INSERM, CHU Limoges, UMR 1092, 2 rue Dr Marcland, 87000 Limoges, France; 2 CHU Limoges, Laboratoire de Bactériologie-Virologie-Hygiène, National Reference Center for Herpesviruses (NRHV), 2 avenue Martin Luther King, 87000 Limoges, France

**Keywords:** cytomegalovirus, DNA packaging, terminase, letermovir

## Abstract

Human cytomegalovirus (HCMV) is responsible for life-threatening infections in immunocompromised individuals and can cause serious congenital malformations. Available antivirals target the viral polymerase but are subject to cross-resistance and toxicity. New antivirals targeting other replication steps and inducing fewer adverse effects are therefore needed. During HCMV replication, DNA maturation and packaging are performed by the terminase complex, which cleaves DNA to package the genome into the capsid. Identified in herpesviruses and bacteriophages, and with no counterpart in mammalian cells, these terminase proteins are ideal targets for highly specific antivirals. A new terminase inhibitor, letermovir, recently proved effective against HCMV in phase III clinical trials, but the mechanism of action is unclear. Letermovir has no significant activity against other herpesvirus or non-human CMV. This review focuses on the highly conserved mechanism of HCMV DNA-packaging and the potential of the terminase complex to serve as an antiviral target. We describe the intrinsic mechanism of DNA-packaging, highlighting the structure-function relationship of HCMV terminase complex components.

## Teaser

Human cytomegalovirus (HCMV) is responsible for life-threatening infections in immunocompromised individuals and can cause serious congenital malformations. Available antivirals target the viral polymerase but are subject to cross-resistance and toxicity. New antivirals targeting other replication steps are therefore needed. DNA-packaging are performed by the terminase complex. A new terminase inhibitor, letermovir, recently proved effective against HCMV in phase III clinical trials, but the mechanism of action is unclear. This review focuses on the highly conserved mechanism of HCMV DNA-packaging and the potential of the terminase complex to serve as an antiviral target.

## INTRODUCTION

Human cytomegalovirus (HCMV) belongs to the betaherpesviruses. It has a double-stranded DNA genome of approximately 230 kb encoding over 200 proteins. Like other members of this subfamily, its main characteristics are high species specificity, various cellular targets and slow replication in cell culture. HCMV persists in a latent state after primary infection and is able to manipulate the immune system by expressing a large number of proteins. In healthy individuals, primary infection is usually asymptomatic or provokes only a self-limited febrile illness. Viremia is rapidly controlled by cell-mediated immunity, but HCMV establishes life-long latency in various cells. Viral reactivation can lead to life-threatening complications in immunocompromised individuals. Despite significant improvements in diagnostic and therapeutic management, cytomegalovirus (CMV) remains a significant problem in immunocompromised individuals, including solid-organ and hematopoietic stem cell transplant recipients and human immunodeficiency virus (HIV)-infected patients (Torres-Madriz and Boucher 2008). HCMV is also the most common infectious cause of congenital malformations, with developmental delay, sensorineural hearing loss and fetal death in 10%–15% of cases. The only drugs licensed for the treatment of HCMV infection and disease are ganciclovir (GCV, Cymevene®), valganciclovir (VGCV, Valcyte®), cidofovir (CDV, Vistide®) and foscarnet (FOS, Foscavir®), all of which target the viral polymerase pUL54. Acyclovir is approved for the prevention of HCMV infection in the European Union (EU). Limitations of these antivirals are their dose-limiting toxicity and resistances emergence. Resistance mutations occur in the *UL97* kinase (GCV) or in the *UL54* polymerase, leading to various levels of cross-resistance to all available antivirals (Lurain and Chou 2010; Chou 2015a; Chaer, Shah and Chemaly 2016). Recent attempts to develop new anti-HCMV compounds have focused mainly on novel targets such as the viral kinase *UL97* (maribavir) and the viral terminase complex involved in viral DNA cleavage/packaging. Several molecules targeting terminase proteins have been discovered (2-bromo-5,6-dichloro-1-beta-D-ribofuranosyl benzimidazole (BDCRB), GW275175X and BAY 38-4766) (Underwood *et al.*^1998^; Reefschlaeger *et al.*^2001^; Williams *et al.*^2003^; Dittmer *et al.*^2005^) but none has reached phase 2 or 3 clinical development, mainly owing to poor bioavailability. Letermovir (AIC246) is derived from a new chemical class, the quinazolines (Lischka *et al.*^2010^), and acts via a novel, not fully understood mechanism involving the viral terminase protein pUL56 (Goldner *et al.*^2011^). Wildum, Zimmermann and Lischka observed no antagonistic effects during letermovir combination with current polymerase inhibitors or with anti-HIV drugs (Wildum, Zimmermann and Lischka 2015). Moreover, Wang and collaborators found that a hydroxypyridonecarboxylic acid compound, previously reported to inhibit HIV RNase H, also inhibited pUL89 at low concentrations (Wang *et al.*^2016^). pUL89 may thus present a potential drug target for HCMV. Here, we review the structure and function of HCMV terminase complex proteins, as new antiviral targets.

### Genome cleavage/packaging, a highly conserved mechanism

Viruses generally use one of two main strategies for genome packaging: the virus either assembles the capsid around the genome (e.g. HIV) or packs the genome into a preformed procapsid. Double-stranded DNA viruses like tailed bacteriophages and herpesviruses use the latter strategy. Herpesviruses and most bacteriophages produce large concatemers during genome replication and then require a motor for cleavage/packaging. Knowledge of herpesvirus DNA-packaging is limited. However, many studies have highlighted similarities between bacteriophages and herpesviruses (Baker *et al.*^2005^). The herpes simplex virus 1 (HSV-1) DNA packaging ATPase is highly homologous to the large terminase subunit of bacteriophages (Przech, Yu and Weller 2003). Packaging mechanisms of bacteriophages such as λ, T3 and T7, which cut at specific sites along the concatemer, resemble those of herpesviruses and therefore present a good model for studying herpesvirus genome packaging. The following section describes current knowledge of the terminase complex of HCMV, partly extrapolated from bacteriophages and other herpesviruses.

### Cleavage/packaging and the HCMV terminase complex

**Figure 1.**
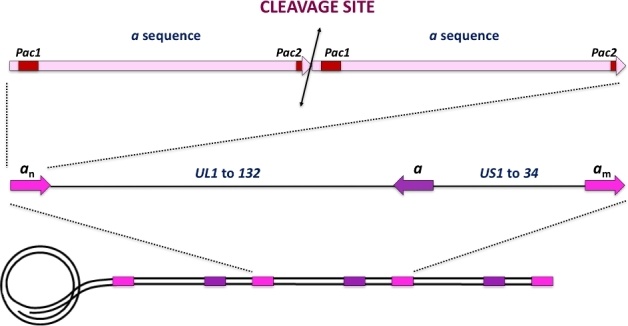
From full genome to cleavage site. Rolling circle replication results in the formation of head-to-tail concatamers that further act as substrates for the DNA-packaging process. The genome is organised as two regions. The unique long (UL) and the unique short (US) segments are flanked by repeated sequences that contain the ≪ *a* ≫ sequence. The pac1 and pac2 sequences are present in each ≪ *a* ≫ sequence.

**Figure 2.**
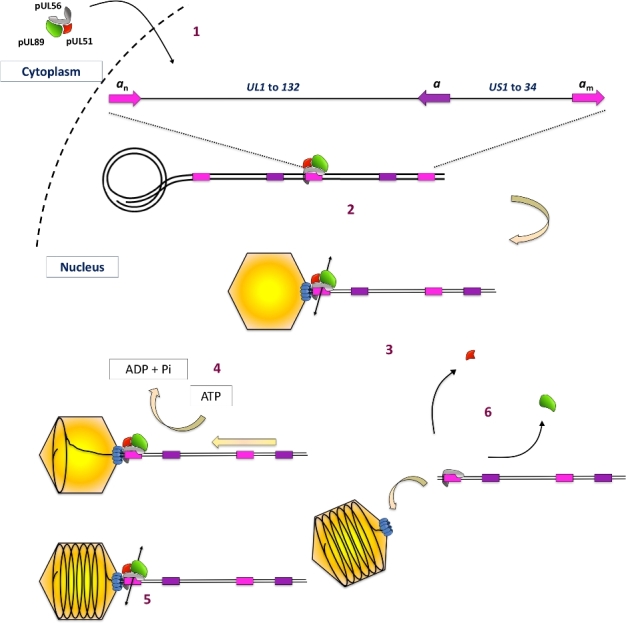
Genome cleavage/packaging and the HCMV terminase complexe adapted from Bogner, Radsak and Stinski (1998). (i) Translocation of the terminase complex into the nucleus, (ii) HCMV terminase specifically binds the pac site and recruits the empty capsid, (iii) cleaves the duplex, (iv) exerts its ATPase activity to power translocation of a unit-length DNA genome into the capsid and (v) completes the DNA-packaging process by cutting off excess DNA at the portal region. (vi) Finally, the DNA-terminase complex dissociates from the filled capsid and is ready for next DNA-packaging step.

As shown for bacteriophage λ, circularisation of the herpesvirus genome occurs early during infection and these circularised molecules act as templates for DNA replication. The viral DNA replicates according to an origin-dependent theta mechanism, in which circular templates are amplified. This step is followed by a rolling circle-based mode of replication that produces concatemers of the genome in head-to-tail fashion; these further act as substrates for the DNA-packaging process (McVoy and Adler 1994). A viral protein complex (terminase) then cleaves concatemeric HCMV DNA into unit-length genomes for DNA packaging. This involves site-specific cleavage at adenine or thymine (AT)-rich core sequences within *pac* motifs (‘*cis-acting packaging signal*’) located in the ‘*a*’ sequence of the terminal and internal repeat segments (Fig. 1). In general, terminase complexes are hetero-oligomers composed of two core proteins (pUL56 and pUL89 for HCMV), each carrying a different function required for the packaging process and associated with several essential cofactors of unknown function. HCMV packaging starts when a packaging signal called the *pac* sequence is recognised on concatemeric DNA by the viral terminase complex. The functional packaging holocomplex, a hetero-oligomer composed of proteins pUL56, pUL89 and pUL51, makes a first specific cut, thus generating a free end at which further packaging is initiated. The DNA/terminase complex then binds to an empty preformed procapsid at its unique portal vertex, across which the DNA is translocated. A second site-specific cleavage step terminates packaging when a unit length genome has been translocated. DNA packaging is followed by cleavage and expulsion of the scaffold protein and angularisation of the capsid. Knowledge of the DNA-packaging process in bacteriophages gave rise to a theoretical model of this process in HCMV (Bogner, Radsak and Stinski 1998). The DNA-packaging steps occur as follows (step numbers refer to respective numbers in Fig. 2): (i) after their translocation into the host cell nucleus, HCMV terminase proteins act to (ii) specifically bind the *pac* site on concatemeric DNA and recruit the empty capsid, (iii) cleave the duplex and (iv) exert ATPase activity to power the translocation of a unit-length DNA genome into the capsid. ATP depletion has been shown to inhibit HSV-1 packaging and lead to the accumulation of B capsid (scaffold containing capsid; Dasgupta and Wilson 1999). (v) The packaging process is completed by cutting off excess DNA at the portal region, leading to C capsids (viral DNA containing capsids). (vi) Finally, the DNA/terminase complex dissociates from the filled capsid and is ready for the next packaging step. Unlike the dsRNA motor, the HCMV terminase complex is a transient component of the viral particle and does not remain in the final virion. The small subunit, pUL89, seems to dissociate shortly after DNA cleavage, as it has never been detected together with pUL56 after this step, and is recycled for further cleavage/packaging (Scheffczik *et al.*^2002^).

### Proteins involved in the DNA cleavage/packaging process

Like dsDNA bacteriophage terminases, the HCMV terminase complex includes a large (pUL56) and a small (pUL89) subunit encoding all the functions of ‘classical’ terminases, such as the processing of viral DNA concatemers. Electron microscopy revealed a toroidal architecture of both proteins, as is commonly the case of DNA-metabolizing proteins (Scheffczik *et al.*^2002^). Further studies suggested that multimers of pUL56 and pUL89 gather to form the oligomeric holoenzyme, but the exact stoichiometry of the complex remains unknown. Additionally, proteins pUL51, pUL52, pUL77 and pUL93 seem to be part of the terminase complex and/or to participate in the DNA cleavage/packaging process (Scheffczik *et al.*^2002^; Borst *et al.*^2013^; Borst *et al.*^2016^; Köppen-Rung, Dittmer and Bogner 2016; DeRussy and Tandon 2015; DeRussy, Boland and Tandon 2016).

### The large terminase subunit, pUL56

**Figure 3.**
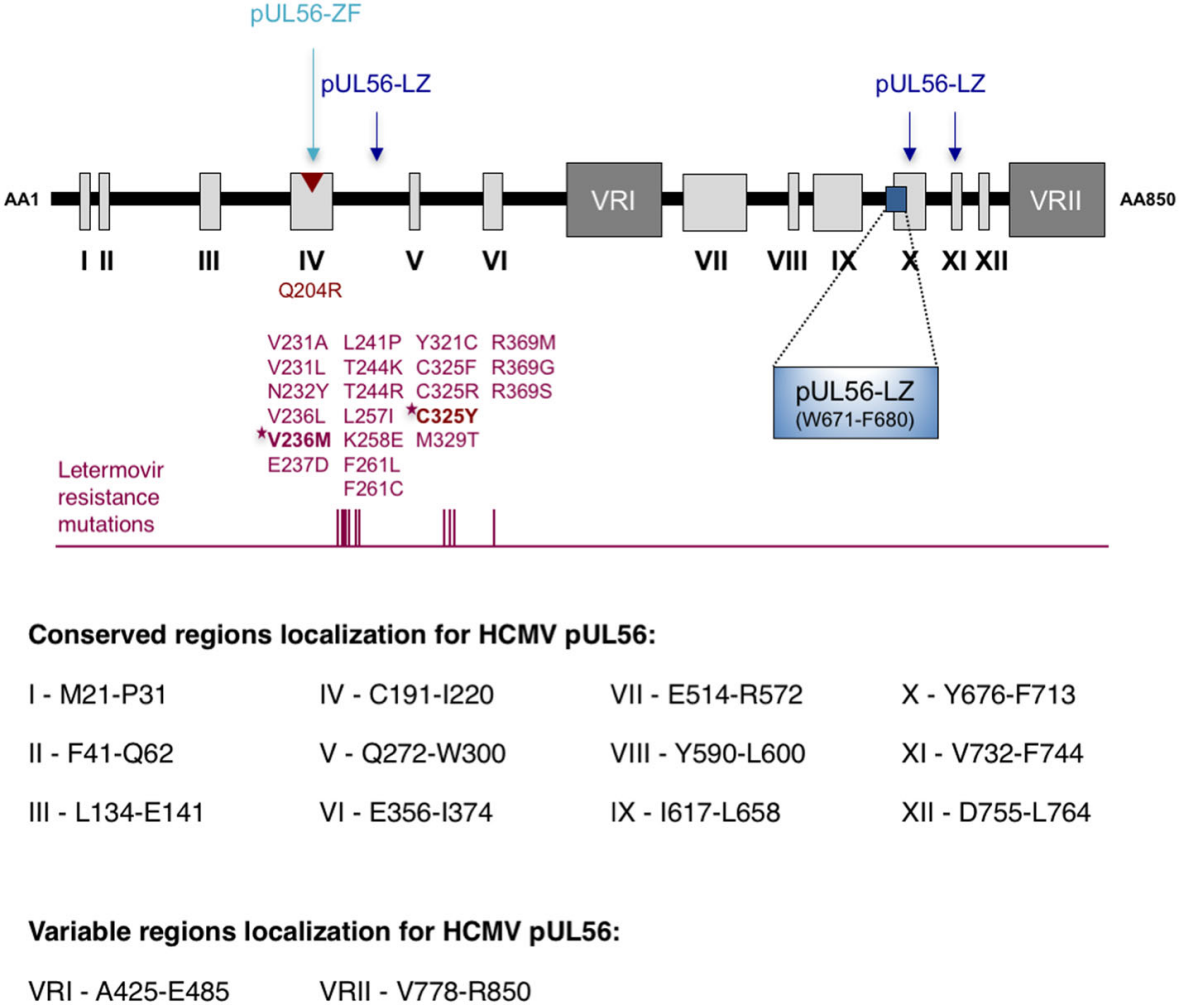
Terminase subunit pUL56 conserved regions adapted from Champier *et al.* (2008). pUL56 is composed of 12 conserved regions (I-XII). The conserved region IV represents the pUL56 zinc-finger domain. The central region of pUL56 and the C-terminus include two variable regions annotated VRI and VRII. The three putative leucine zippers, annotated pUL56-LZ, are indicated. The short sequence in the C-terminal region of pUL56 (_671_WMVVKYMGFF_680_), essential for interaction with pUL89 is highlighted. Positions of amino acids associated with *in vitro* resistance to letermovir are highlighted. Resistance mutations that have been identified in clinical studies are notified in bold with a star. The position of the Q204R benzimidazole resistance mutation is shown in red.

The large subunit of the HCMV terminase complex, pUL56, is composed of 12 conserved regions (I–XII; Champier *et al.*^2008^) and is encoded by ORF *UL56* located on the unique long portion of the viral genome (Fig. 3). This highly conserved protein of approximately 130 kDa was identified and partially characterised through its homology with HSV-1 pUL28, which has an essential role in HSV-1 genome packaging (Bogner *et al.*^1993^; Addison, Rixon and Preston 1990). Three-dimensional reconstruction by electronic cryomicroscopy suggests that pUL56, when expressed alone, exists as a dimer formed by two ring-shaped structures connected to each other by a bridge to their base (Savva, Holzenburg and Bogner 2004). However, no crystal structure has so far been obtained, and its association with the other proteins of the complex has not been fully elucidated. Numerous *in vitro* studies confirmed the activity of pUL56. Using electrophoretic mobility shift assays, Bogner and colleagues demonstrated a sequence-specific interaction of pUL56 with *pac* motifs within ‘*a*’ sequences of the viral genome (Bogner, Radsak and Stinski 1998). A short 128-bp sequence containing regulatory *cis* elements in conjunction with *pac* motives is sufficient to mediate efficient HCMV genome maturation (Wang and McVoy 2011). Initiation of the DNA-packaging process takes place in nuclear structures known as replication centres, where pUL56 accumulates at late times post infection and co-localises with pUL112-113 and pUL44, three proteins involved in viral replication. Nuclear importation of pUL56 is mediated by an importin-dependent pathway through the interaction of hSRP1a with the C-terminal nuclear localisation signal (NLS) of pUL56 (amino acid residues 816–827). Alanine scanning identified arginine 822 and lysine 823 as the essential residues of the pUL56 NLS for nuclear translocation (Giesen, Radsak and Bogner 2000). Whereas pUL56 translocates into the nucleus when expressed alone, correct nuclear localisation of both pUL89 and pUL51 requires the concurrent presence of all three terminase subunits (Neuber *et al.*^2017^). pUL56 and pUL89 play a major role in driving the complete DNA cleavage and packaging process. Co-immunoprecipitation experiments performed by Hwang and Bogner detected a specific interaction between pUL56 C-terminal and pUL89, as confirmed by Thoma and colleagues (Thoma *et al.*^2006^). Recently, we have shown that a short sequence in the C-terminal region of pUL56 (_671_WMVVKYMGFF_680_) is essential for interaction with pUL89 (Ligat *et al.*^2017^). Furthermore, a recent study has shown a mutual interplay between terminase subunits pUL56, pUL89 and pUL51 as a prerequisite for terminase assembly and nuclear localisation (Neuber *et al.*^2017^). The first step during the cleavage of concatemeric DNA catalysed by pUL89 is an essential one and is followed by the binding of the terminase complex to a *pac* sequence. It is generally accepted that pUL56 acts as an ‘anchor’ for pUL89. Some studies indicate that pUL56 has ATP-independent endonuclease activity that seems to be *pac* specific (Bogner, Radsak and Stinski 1998). Moreover, pUL56 could enhance the endonuclease activity driven by pUL89 (Scheffczik *et al.*^2002^). pUL56 also interacts with the viral portal protein pUL104 during DNA-packaging via the C-terminal part of pUL56. This interaction is crucial during the DNA-packaging process: its prevention by the benzimidazole-D ribonucleosides BDCRB and 2,5,6-trichloro-1-beta-D-ribofuranosyl benzimidazole (TCRB) inhibits HCMV maturation (Krosky *et al.*^1998^; Dittmer *et al.*^2005^). More importantly, the viral DNA-packaging process is energy-dependent and requires terminase ATPase activity. *In vitro* studies of bacteriophages have shown that ATP hydrolysis, generating one ATP molecule, allows DNA-packaging of two base pairs (Guo, Peterson and Anderson 1987). In almost all bacteriophages, the large terminase subunit catalyses ATP-dependent translocation of genomic DNA to the proheads (Rao and Feiss 2015). Hwang and Bogner demonstrated that, in HCMV, the terminase ATPase activity is only associated with pUL56. pUL56 ATPase activity is enhanced by up to 30% when it is associated with pUL89 (Hwang and Bogner 2002). A similar mode of action has been documented for the phage T4 terminase complex, with the subunit gp16 enhancing the ATPase activity of the subunit gp17 (Leffers and Rao 2000). Site-directed mutagenesis indicates that the glycine and lysine at positions 714 and 715, respectively, of the putative ATP binding site _709_YNETFGKQ_716_ are essential for ATP hydrolysis (Scholz *et al.*^2003^). The pUL56 Q204R substitution associated with BDCRB and TCRB resistance is located within a putative zinc finger, implicating this region in the benzimidazole d-ribonucleoside mechanism of action (Champier *et al.*^2008^) (Krosky *et al.*^1998^) while mutations conferring resistance to letermovir are located in a non-conserved region. Q204R does not confer resistance to letermovir (Lischka *et al.*^2010^; Chou 2015b; Fig. 3). This underlines the different mechanisms of action of these drugs and the need to further explore the possible functional roles of these domains.

### The small terminase subunit, pUL89

**Figure 4.**
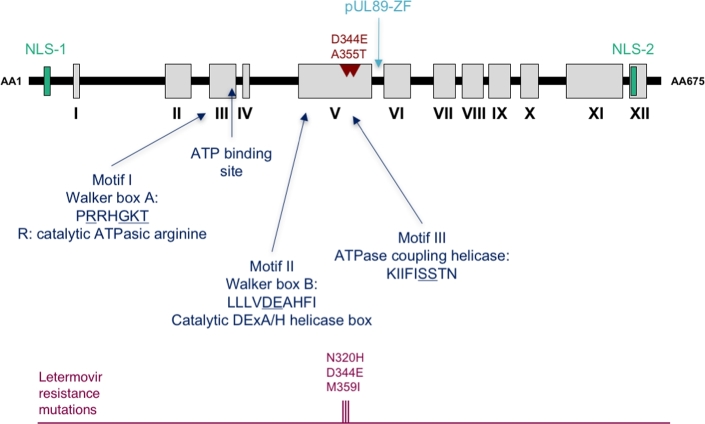
Terminase subunit pUL89 conserved regions adapted from Champier *et al.* (2007). pUL89 is composed of 12 conserved regions (I-XII) with several putative functional domains such as nuclear localisation site (NLS-1 and NLS-2), pUL89 zinc-finger domain annotated pUL89-ZF, adenine binding site (Walker A box, Walker B box and ATPase coupling helicase, ATP binding site. Underlined amino acids are residues involved in the activities of domains. Positions of amino acids associated with *in vitro* resistance to letermovir are highlighted. The position of the D344E and A355T benzimidazole resistance mutations are shown in red.

The small subunit of the HCMV terminase complex is encoded by ORF *UL89*, located on the long unit of the viral genome. *In vitro* translation and eukaryotic expression demonstrated that pUL89 is a 70- to 75-kDa protein in monomeric form, and 150 kDa when dimerised (Hwang and Bogner 2002; Thoma *et al.*^2006^). pUL89 is a homolog of HSV-1 protein pUL15 and was initially identified as a terminase subunit of the HCMV terminase complex by Hwang *et al.* A previous study by Davison had shown similarities in the amino acid sequence of HCMV pUL89 and the terminase subunit gp17 of phage T4. Because of the strong homology of part of pUL89 to the ATP binding motif of the bacteriophage T4 gp17 subunit, the possible role of pUL89 in HCMV DNA-packaging was investigated (Davison 1992; Hwang and Bogner 2002). Subsequently, our *in silico* study (Champier *et al.*^2007^) focusing on the amino acid sequence of pUL89 have highlighted the four motifs involved in the ATPase centre domains located in N-terminal part of pUL89: the adenine binding site (_156_EPFQ_159_ in HCMV), the Walker A box or motif I (_213_PRRHGKT_219_ motif in HCMV), the Walker B box or motif II (_305_LLLVDEAHFI_314_ in HCMV) and motif III (_337_SST_339_ in HCMV pUL89) (Champier *et al.*^2007^; Fig. 4). These motifs have also been identified by Mitchell *et al* in the terminase subunit of the bacteriophage T4 protein gp17 (Mitchell *et al.*^2002^). Despite its partial homology with the terminase subunit of T4 gp17, HCMV pUL89 did not exhibit enzymatic ATPase activity. However, it enhanced pUL56-associated ATPase activity described in the previous paragraph by about 30%, indicating a direct interaction of pUL89 with pUL56 (Hwang and Bogner 2002; Leffers and Rao 2000). *In vitro* binding assays using Glutathion-S-Transferase (GST) fusions, an HCMV-Δ*UL89* mutant and BAC complementation experiments indicated that the 580–600 domain of pUL89 was necessary to bind with pUL56 (Thoma *et al.*^2006^). This short domain was then studied *in silico* and its structure was resolved as an alpha helix (Couvreux *et al.*^2010^). The crystal structure revealed that the pUL89 C-terminal region corresponds to an exposed helix with three fully conserved residues (Lys583, Ala586 and Asn595), probably forming the interaction domain between pUL56 and pUL89 (Nadal *et al.*^2010^). This interaction likely takes place in the cytoplasm, after which the terminase proteins are translocated to the nucleus. Two putative NLS have been proposed to catalyse the nuclear translocation of pUL89 (Champier *et al.*^2007^). Recent findings indicate that the pUL89 subunit translocates to the nucleus only in presence of pUL56 and pUL51, and otherwise remains exclusively in the cytoplasm (Wang *et al.*^2012^; Neuber *et al.*^2017^). Both HCMV terminase subunits were found to have random nuclease activity *in vitro*. Nevertheless, it has been suggested that pUL56 is unable to exert specific cleavage by itself and that, once again, synergy with pUL89 is necessary to complete the cleavage steps of the DNA-packaging process during HCMV replication (Scheffczik *et al.*^2002^). Structural data obtained by Nadal *et al.* indicate that the pUL89 C-terminal domain belongs to the RNase H-like superfamily of nuclease and polynucleotidyl transferases. Indeed, it has the characteristic fold of this superfamily, and three conserved acidic residues (Asp463, Glu534 and Asp651) coordinating two Mn^2+^ cations. The D344E and A355T substitutions in pUL89 are associated with BDCRB and TCRB resistance, implying that pUL89 is also involved in the mechanism of action of benzimidazole D-ribonucleosides (Krosky *et al.*^1998^; Underwood *et al.*^1998^).

### Other proteins of the terminase complex

DNA-packaging is probably more complex for herpesviruses genomes than for bacteriophages, and seems to involve more proteins than the terminase subunits pUL56 and pUL89. Based on data obtained with alphaherpesvirus mutants, it was suggested that, besides the genuine terminase subunits pUL56 and pUL89, at least four additional HCMV proteins, namely pUL51, pUL52, pUL77 and pUL93, contribute to this process. These proteins are homologous to the HSV-1 proteins pUL33, pUL32, pUL25 and UL17, respectively (Borst *et al.*^2008^; Borst *et al.*^2013^; Borst *et al.*^2016^; Köppen-Rung, Dittmer and Bogner 2016; DeRussy and Tandon 2015; DeRussy, Boland and Tandon 2016). Knowledge of these HSV-1 proteins is limited. HSV-1 pUL33 interacts with the HSV-1 terminase proteins pUL15 and pUL28 and with the portal protein pUL6 (Beard, Taus and Baines 2002). Although HSV-1 pUL32 is required for both DNA cleavage and packaging, HSV-1 pUL25 is not required for DNA cleavage but is necessary for efficient DNA-packaging (McNab *et al.*^1998^). Recent findings indicate that knockdown of HCMV pUL51 results in the absence of DNA-filled capsids (C capsids) in infected cells, suggesting a role of pUL51 in DNA-packaging. pUL51 interacts with the HCMV terminase subunits pUL56 and pUL89 and mediates their correct subnuclear localisation (Borst *et al.*^2013^). Moreover, Neuber *et al.* reported that only the fully assembled terminase complex consisting of pUL56, pUL89 and pUL51 is protected from proteasome turnover (Neuber *et al.*^2017^). In pUL52 deletion mutants, viral concatemers remain uncleaved, but pUL52 does not seem to be involved in the nuclear localisation of the pUL56 and pUL89 subunits. Furthermore, contrary to other packaging proteins, pUL52 is not detected in viral replication compartments. Thus, pUL52 might have a distinct function in HCMV DNA-packaging (Borst *et al.*^2008^). pUL93 interacts with pUL77 and components of the nuclear egress complex, namely pUL50, pUL53 and pUL97. Upon knockdown of pUL77 and pUL93, only B capsids are produced, indicating a putative role of these proteins during viral capsid formation (Köppen-Rung, Dittmer and Bogner 2016; DeRussy and Tandon 2015; DeRussy, Boland and Tandon 2016). These findings demonstrate an essential role of these proteins in HCMV DNA-packaging. However, their biochemical makeup and biological functions are poorly characterised. In addition, the three-dimensional structure of these proteins has not yet been resolved (Table 1).

**Table 1.** Overview of terminases homologs of herpesviruses and phages.

	Homologs														
	HCMV			HSV			Phage λ			Phage T4			Phage T3/T7		
Function	Name	Mass (kDa)	References	Name	Mass (kDa)	References	Name	Mass (kDa)	References	Name	Mass (kDa)	References	Name	Mass (kDa)	References
Large terminase subunit binds to DNA pac motifs	*UL56*	130	(Scheffczik *et al.*^2002^), (Bogner *et al.*^1993^)	*UL28*	85	(Addison, Rixon and Preston 1990), (Tengelsen *et al.*^1993^)	*gpNu1*	20	(Becker and Murialdo 1990)	*gp16*	18	(Bhattacharyya and Rao 1994)	*gp18*	20	(Morita, Tasaka and Fujisawa 1995)
Small ATPase subunit of terminase	*UL89*	75	(Champier *et al.*^2007^), (Couvreux *et al.*^2010^)	*UL15*	81	(Yu and Weller 1998), (Przech, Yu and Weller 2003)	*gpA*	73	(Becker and Murialdo 1990)	*gp17*	70	(Bhattacharyya and Rao 1994)	*gp19*	67	(Morita, Tasaka and Fujisawa 1995)
DNA-packaging	*UL51*	17	(Borst *et al.*^2013^)	*UL33*	36	(Beard, Taus and Baines 2002)	NA			NA			NA		
Interacts with terminase	*UL52*	76	(Borst *et al.*^2008^)	*UL32*	150	(Chang, Poon and Roizman 1996)	NA			NA			NA		
Interacts with terminase	*UL77*	100	(Köppen-Rung, Dittmer and Bogner 2016), (Borst *et al.*^2016^)	*UL25*	60	(McNab *et al.*^1998^)	NA			NA			NA		
Interacts with terminase	*UL93*	70	(Köppen-Rung, Dittmer and Bogner 2016), (Borst *et al.*^2016^)	*UL17*	20	(Toropova *et al.* 2011)	NA			NA			NA		

NA: not available

### The HCMV terminase complex as a therapeutic target

Three major drugs, ganciclovir, cidofovir and foscarnet, all targeting the HCMV polymerase pUL54, are routinely used for the prevention and treatment of HCMV infection in the transplant setting. However, the emergence of CMV cross-resistance to available antiviral drugs, favoured by long-term exposure, use of low doses and prolonged immunosuppression, is a growing therapeutic challenge, along with the toxicity of these drugs. Because of their hematologic and nephrologic toxicity, these drugs are not recommended for use in pregnant women. The HCMV terminase complex is a critical component of the DNA-packaging process that translocates viral DNA into the empty capsid. The large subunits pUL56 and pUL89 have an essential role in this process, containing many of the functional sites required for DNA-packaging. Nevertheless, little is known of other proteins that belong to the terminase complex. Knowledge of the interactions between the HCMV terminase subunits can serve as a starting point for the generation of new antivirals that target the interaction between these key viral proteins. The terminase complex is highly CMV-specific, with no counterpart in mammalian cells, and thus represents a target of choice for new antivirals. This has been confirmed by the recent development of letermovir in the transplant setting (Lischka *et al.*^2010^). Preclinical data suggested that letermovir targets pUL56 (Lischka *et al.*^2010^). It is a potent antiviral with *in vitro* activity surpassing the current gold standard, GCV, by over 400-fold for the 50% effective concentration (EC50; 4.5 nM versus 2 μM) and over 2000-fold the EC90 (6.1 nM versus 14.5 μM), without significant cytotoxic effects (Marschall *et al.*^2012^; Goldner *et al.*^2011^; Lischka *et al.*^2010^). In phase II trials, letermovir effectively prevented HCMV infection in recipients of allogeneic hematopoietic cell transplants, with an acceptable safety profile (Chemaly *et al.*^2014^). The phase III trial started in 2014 (by Merck Sharpe and Dohme Corps) was completed at the end of 2016 (ClinicalTrials.gov Identifier: NCT02137772), and published in 2017 (Marty *et al.*^2017^). Letermovir significantly reduced the rate of clinically significant infection at week 24 post graft (end-organ disease or initiation of anti-HCMV pre-emptive therapy based on viremia). And was overall well tolerated. Under the name of Prevymis, letermovir has been recently approved (august 2017) by the US Food and Drug Administration as a new molecular entity for prophylaxis of cytomegalovirus infection and disease in adult CMV-seropositive recipients of an allogeneic hematopoietic stem cell transplant (www.accessdata.fda.gov, Reference ID 4179078). As transplant recipients receive antivirals coadministered with cyclosporine A (CsA) or tacrolimus (TAC) as immunosuppressants, clinical trials investigated the potential for letermovir–immunosuppressant interactions. Letermovir increased CsA and TAC exposure. Contrary to TAC, CsA altered letermovir pharmacokinetics (Kropeit *et al.*2017b). Hepatic and renal impairment also affect letermovir pharmacokinetics. Moderate hepatic impairment increases letermovir exposure less than 2-fold, and severe hepatic impairment 4-fold (Kropeit *et al.*2017a). Renal impairment also increases letermovir exposure (Kropeit *et al.*2017c) (www.accessdata.fda.gov, Reference ID 4179078).

*UL89* and *UL56* mutations are known to confer benzimidazole resistance. A large number of letermovir resistance mutations in *UL56* that have been identified *in vitro* or in clinical studies, clustered at *UL56* codons 231–369 (Fig. 3). Thus, these mutations were located outside the functional domains of pUL56 involved in DNA-packaging and do not impact viral replicative capacity (Goldner *et al.*^2014^; Chou 2015b); Lischka, Michel and Zimmermann 2016). Moreover, letermovir occasionally selected *UL89* N320H, D344E and M359I mutations *in vitro* (Chou 2017a). After culture of HCMV laboratory strain under letermovir, P91S mutation in gene *UL51* was observed in 2 experiments. P91S alone conferred a letermovir resistance and when combined, multiplied letermovir resistance conferred by *UL56* mutations S229F or R369M (Chou 2017b). The cross-resistance mapping to 3 genes suggests that letermovir is targeting a mechanism that depends of the interaction of pUL56, pUL89 and pUL51. The impact of the *UL56* V236M mutation, alone and combined with *UL54* E756K, on drug susceptibility and the replicative capacity of recombinant HCMV was recently evaluated. The V236M and E756K double mutant exhibited borderline resistance to current antivirals and letermovir and replicated less efficiently than the wild-type virus *in vitro* (Piret, Goyette and Boivin 2017).

Emergence of resistance in the clinical studies has not been fully documented yet. Few patients experienced resistance during prophylaxis failure. In the phase II study only a short sequence including *UL56* codons 231–369 has been sequenced. One out of 12 patients with prophylaxis failure had a pUL56 V236M substitution. In the phase III study, the entire *UL56* and *UL89* genes were sequenced. Three out of 28 patients who failed prophylaxis had pUL56 V236M, C325W and E237G substitutions during treatment (www.accessdata.fda.gov, Reference ID 4179078). However, the clinical significance of C325W and E237G substitutions is not known to date (www.accessdata.fda.gov, Reference ID 4179078).

Unlike other viral DNA-packaging inhibitors, letermovir is remarkably specific for HCMV (Table 2; Marschall *et al.*^2012^). Marschall *et al.* demonstrated potent *in vitro* activity of letermovir against 17 HCMV clinical isolates and no significant activity against any other herpesvirus. These findings point to a mechanism of action distinct from that of other DNA-packaging inhibitors, which are less specific and less efficient than letermovir. Although broad anti-herpesvirus activity would be a plus, the potential importance of letermovir, as a safe, specific and potent candidate antiviral, cannot be overstated, especially in view of the poor safety profile of drugs currently approved for prevention or treatment of HCMV infection (Griffiths and Emery 2014).

**Table 2.** Antiviral activity of letermovir against alpha-, beta- and gammaherpesviruses adapted from Marshall *et al.* (2012).

Virus (strain)	AIC246 EC_50_ ± SD (μM)^a^
Alphaherpesviruses	
VZV (Oka)	>10
HHV_1 (166v VP22-GFP)	>10
HHV_2 (01-6332)	>10
Betaherpesviruses	
HCMV (AD169-GFP)	0.0027 ± 0.0002
MCMV (Smith)	4.5 ± 2.0
RCMV (Maastricht)	>10
HHV_6 (A-GS)	>10
Gammaherpesviruses	
EBV (B95-8)	>10

a EC_50_ values were determined by specific cell culture-based antiviral test systems. Data are means of results from at least three independent experiments and are expressed with standard deviations.

### Concluding remarks

The HCMV terminase complex is highly herpesvirus specific and has no counterpart in mammalian cells. It thus represents a target of choice for antiviral drug development. A better understanding of the HCMV DNA-packaging process, together with the structure and function of the necessary components, will enable the development of antivirals with high specificity and low toxicity. Likewise, elucidation of the letermovir mechanism of action will hasten the development of new terminase inhibitors, not only in HCMV but also in other herpesviruses. Even if letermovir has no activity against herpesviruses other than HCMV, the conserved amino acid sequences of pUL56 and its homologues of other herpesvirus suggest that letermovir derivatives may be active against other herpesviruses. Finally, combining drugs such as letermovir with available pUL54 polymerase inhibitors could hold potential for the treatment of HCMV infection.
